# Mesmerism, Galvanism and Quackery

**Published:** 1983-04

**Authors:** P. S. Brown

**Affiliations:** Reader in Pharmacology, University of Bristol


### Mesmerism, galvanism and quackery

**P. S. Brown**, B.M., B.Ch., M.R.C.P.
Reader in Pharmacology, University of Bristol

In the middle decades of the nineteenth century the British medical profession was attempting to organise its ranks and define its boundaries. The separation of the unqualified from the qualified practitioners in Bristol has already been discussed[1], but even within the profession it was necessary to define orthodoxy and discourage 'quackery'. The most frequently quoted medical heresies were homoeopathy, hydropathy and mesmerism[2], but there were many others. An activity which did not attract such medical condemnation, however, was 'medical galvanism', a descriptive term often used loosely to mean treatment by electricity however applied, e.g., by 'drawing sparks' from the affected part using the charge from a 'frictional machine' (Franklinism) or causing muscle twitches by placing contacts on the skin over muscle or nerve and applying interrupted current from batteries (Galvanism) of an induction machine (Faradism). Both mesmerism and medical galvanism were well represented in Bristol in the 1840s and '50s, and it is instructive to ask why one was vigorously rejected and one was not.

### MESMERISM

At the beginning of the century, George Winter[3] wrote an account of Mainauduc's lectures on mesmerism in Bristol in 1788–89, and Munro Smith has described some sniping at Edward Long Fox sen. for his passing interest in mesmerism.[4] In the middle decades of the nineteenth century the Bristol newspapers made frequent mention of mesmerism or animal magnetism as it was also called. Visiting lecturers and 'mesmeric performers'[5] included those described as Dr. Cantor, Mr. H. Brookes, Mr. W. J. Vernon, Dr. Owens MRCS, Mr. Lundie, Spencer T. Hall, Mr. Barnett (from America) and Adolphe, frère d'Alexis. The most active mesmerists resident in Bristol at this time were Samuel D. Saunders, who was not medically qualified, and Henry Storer, LSA and MD (Aberdeen), both of whom had previously lived in Bath. One other person calling himself a mesmerist was found in the census returns for 1851 but has not been traced otherwise.

Storer lectured and wrote on mesmerism[6], trying to concentrate attention on its medical uses. He became physician to the Bristol Mesmeric Institute, at 10 Park Street, which was founded in 1848 after exploratory private meetings.[7, 8] The Institute, with the Earl Ducie as president and Dr. Elliotson FRS as nominal consulting physician, had a quarter-page entry in the 1852 *Bristol Directory*, immediately below that for the Bristol Medical School. But reports of its annual meetings suggest that there was a continuing lack of financial support[9] and the Mesmeric Institute seems to have collapsed in 1852. Editorial comment in the mesmeric journal, *Zoist*, ascribed its failure to 'dissension and mismanagement'.[10]

The inevitable medical opposition to mesmerism in Bristol was led by John Bishop Estlin, FRCS, who was disturbed by lectures on mesmerism being given at the Bristol Institution. In 1843, he urged his colleagues not to become involved in examining mesmeric phenomena. He was sceptical of much that the mesmerists claimed and attributed the rest to 'the power of the imagination'. He condemned the impropriety of 'bringing forward young females before large audiences as the subjects of experiment', concluding that the medical profession should protect the minds of those around them from 'delusive speculations in misnamed sciences'.[11] Estlin returned to the attack in 1845, noting that the exhibitors of mesmeric phenomena were usually 'of a class below our own' and motivated to practise deception.[12]

Another medical spokesman commenting on mesmerism was John Addington Symonds, the prominent Bristol physician. Speaking at the Bristol Institution in 1851, he referred to animal magnetism, mesmerism, electro-biology, electro-psychology, ethero-biology and such like as 'dubious regions, dimly-lighted, phantom-haunted' for exploring which he had 'little leisure, less inclination, and certainly no vocation'.[13] Both Symonds and Estlin were members of the small but active group drawn from Bristol's educationalists, leading nonconformists and members of the medical profession which has been identified by Mellor as crucial in the promotion of cultural institutions in the city.[14] Their views carried weight.

### GALVANISM

The medical galvanists practising in Bristol during the 1840s and '50s were more numerous than the mesmerists. Sixteen individuals calling themselves medical electricians or galvanists have been identified from the sources described in previous studies.[15] The scope of their activities is indicated by the following advertisement of William Hazard: other galvanists professed to treat a wide range of conditions but Hazard's list is particularly comprehensive.

'*Diseases which come under the curative power of Electricity and Galvanism, are the following*: Hypochondriasis, Hysteria, St. Vitus's Dance, Paralysis (under certain Pathological Conditions), such as, from Poison of Lead, Dropped Hands, Rheumatic Paralysis; the Facial Nerve (portio dura) following local Injury, Hysterical, Loss of Nervous Power, and Sensation, etc. (but not when permanent irritation exists in the brain); Fainting, Giddiness in the Head, Trembling, Lock-jaw, Cramps, Palpitation, Asthma, Angina Pectoris, Tic Douloureux, Headache, General Debility, Epilepsy, Indigestion, Toothache, Constipation, Stricture of the Rectum (spasmodic or permanent), Liver Complaint (acute or chronic), Gout, Rheumatism, Lumbago, Spinal Complaint, Sciatica, Flatulency, Want of Appetite, Amaurosis, Weak Eyes, Deafness, Stammering, Loss of Voice, Smell and Taste, Cold Hands and Feet, (Female Complaints, Uterine Inertia, Amenorrhoea and Dysmenorrhoea), Asphyxia, Narcotic Poisoning, Suspended Animation in cases of Drowning, etc., etc., Mr. H. was for many years a Student in the Hospital of La Pitie, Paris, and also a pupil of the late Dr. Riley, Bristol. *No Fee required for Consultations*'.[16]

Hazard claimed some medical experience though previously he had been a teacher of drawing. Another Bristol galvanist with some medical background was James Butcher who practised from 1848 as an unqualified 'surgeon', but by 1858 had dropped this designation and become a chemist, dentist and galvanist.[1] The other person with known medical connections was Edward Maish who had been the dispenser at Bristol General Hospital and thereafter appeared in directories as a chemist, cupper, surgeon dentist and medical galvanist.

Among the other galvanists were three members of the Randall family which was associated with both astrology and herbalism[15], and two successive proprietors of a mechanical surgery, dealing in appliances such as trusses, and announcing in advertisements that medical electricity was administered 'from a powerful Machine, and Galvanism medically applied from an Electro-Magnetic Apparatus'. Another was F. W. Griffin, proprietor of the Bristol School of Chemistry, who also called himself a medical electrician, administering 'frictional electricity'.[17] The group also included a medical galvanist and apparatus maker who was active in Bristol for a short time only, as were a representative of the firm of J. & H. Newton of London and Birmingham, and a chemist and druggist who also practised galvanism. The partnership of Smart & Osborne was similarly short-lived but J. Sherrard Smart continued alone as an active 'galvanist and lecturer on medical application of electricity': later he became a surgeon dentist.

The two remaining galvanists were directly associated with mesmerism. George Gore, active in Bristol from 1847 to 1850, advertised as a professor of phrenology and medical galvanism, ending his advertisement with the phrase – 'Mesmerism medically applied'[18]: and Saunders, after practising for nearly ten years in Bristol as a mesmerist, made his final appearance in the Directory of 1861 also as a medical galvanist. William Hazard, mentioned above, assisted in some of Saunders' mesmeric demonstrations and wrote to the newspapers supporting the suggestion of providing mesmeric facilities in the local hospitals, referring to himself as an amateur mesmerist.[19]

### MEDICAL ATTITUDES

The medical profession objected to the mesmerists not only because their theory conflicted with accepted medical theory but also for social and professional reasons, as has been pointed out by Parssinen.[20] Many of the mesmerists had therapeutic aspirations but no medical training and were seen as grossly ignorant by the profession. Some gave highly dramatic performances, more as entertainment than serious communication, and were believed to obtain their effects by deception. Performances might include mesmerising a young female and demonstrations of clairvoyance, sometimes used for medical diagnosis. And the mesmerists were financial competitors in that they were patronised by the well-to-do whose interest they aroused and whose involvement they won by their lecture-demonstrations.

At first sight, the galvanists might seem to have been objectionable to the medical profession for several of the same reasons. They were sometimes directly associated with mesmerism and current reports in the national press linked them with other forms of what the regular practitioners saw as quackery. William Hooper Halse advertised as a medical galvanist and proprietor of the Galvanic Family Pill, and had given demonstrations of mesmerism.[21] Dr. De l'Huynes published a flimsy journal called *Medical Galvanism*, advertising his treatment both by galvanism and by eclectic medicine which embraced hydropathy, homoeopathy and many other systems.[22] The use of medicines as well as electricity was probably common; in Stafford, an itinerant medical galvanist was found guilty of manslaughter by administering colchicum without due care[23] and, in Leeds, a medical galvanist was a leading supporter of the medical botanist John Skelton, a frank opponent of the regular practitioners[24]. And stirred by these reports must have been memories of the use since the previous century of electrical treatment by many irregular practitioners, including the spectacular quackery of James Graham, famed for his celestial bed.[25]

The medical galvanists could also be seen as financial competitors: half of those identified practised in the rich north-western quadrant of Bristol (as defined previously[26]). Admittedly the public were not involved in the same way as they were in mesmerism, though they were encouraged to purchase apparatus for self-treatment and there were some public lectures on galvanism. As already noted, Smart called himself a lecturer, and lectures were given in Bristol by Mr. Hicks, a medical galvanist from Bath.[27]

Despite these activities of the mid-nineteenth century galvanists, the medical profession's reaction to them was not vociferous. Certainly there were some comments: the *Lancet* carried a scathing review of a book by Lawrance advocating galvanism[28], and published a case report which appeared to link galvanism with mesmerism and label both as charlatanry.[29] But professional acceptance of galvanism was going ahead. In 1836 an electrical room had been established at Guy's Hospital by Golding Bird, who had lectured on galvanism at the Royal College of Physicians, and in subsequent decades its use increased in other London hospitals.[30] Galvanism also made progress in Bristol where physicians like J. A. Symonds, who had had no time for mesmerism, advised electrical treatment for selected conditions.[31] But there were some who had reservations and, in 1882, the Bath physician John Kent Spender still detected 'an unquestionable scepticism' about the therapeutic application of electricity.[32] But by 1901, George Parker had been designated as physician in charge of the Electrical Department of Bristol General Hospital[33] and wrote describing its work and equipment.[34]

Why then was galvanism accepted, even if reluctantly by some, while mesmerism was rejected at this time? The crucial distinction was probably that the effects of electricity differed from those of the supposed mesmeric influence in being easily and convincingly demonstrable, and by having a predominantly somatic manifestation. Study of this obvious effect on nerve and muscle seemed to run parallel to impressive progress in basic physical science and offered a mode of intervention in bodily function that was irresistable as a potential therapy. While many authors considered that the medical application of electricity had not yet had a fair trial, most would have agreed with an editorial in the *Lancet* which concluded that 'we cannot persuade ourselves that an agent which so powerfully affects the nervous system . . . would not, if we knew how to handle it properly, admit of very important applications to the treatment of disease'.[35]

So galvanism was acceptable to the medical profession on theoretical grounds, but there still remained the problem that the mass of its practitioners were not medically qualified. Letters in the *Lancet* in 1859 warned that 'unqualified electricians' might evade the new registration act[36] and, as already mentioned, one unqualified 'surgeon' in Bristol did move into medical galvanism at about this time.[1] The status of galvanism was clarified by court actions in 1861 when a 'medical and operative galvanist' named Thiselton sued a defaulting client for fees. Not being medically qualified he was not registered and therefore not entitled to recover charges for medical attendance. His council contended that the galvanic operations were no more medical or surgical operations 'than were the cutting of corns and toenails and the administration of Turkish baths'. This view was eventually accepted and medical galvanism was legally categorised as merely ancillary to medical practice.[37]

Most of the galvanists were probably content to accept this implied status. Smart and Osborne, when advertising their new apparatus, had thanked 'those Medical Gentlemen who have already liberally and disinterestedly sanctioned the application of Galvanism in connexion with their patients':[38] and a chemist and medical electrician in Bath, when publishing a collection of his successful cases, noted that the application of electricity had been 'with the advice of the medical attendants'.[39] Gore described himself as medical galvanist 'to the Faculty' just as surgeon dentists sometimes used the phrase to suggest their approval by the medical profession. Recognition of the galvanists' status was reflected in *Mathews' Bristol Directory*. Prior to 1851 the professional lists were under headings for physicians, surgeons, surgeon dentists and veterinary surgeons. In 1851 a new heading for medical galvanists was added.

### REFERENCES*

1. BROWN, P. S. (1981) Defining the medical profession in mid-nineteenth century Bristol, *Bristol Med.-Chir. J.*, **96**, 4–7.
2. *Br.for.med.Chir.Rev.*, 1850, **5**, 285–310.
3. WINTER, G. (1801) History of animal magnetism; its origin, progress and present state, London, Newberry.

* I am grateful to Avon County Library for access to local source material and to the staff of the Bristol Central Reference Library for locating and producing the frequently cumbersome items.

4. SMITH, G. M. (1917) A history of the Bristol Royal Infirmary, Bristol, Arrowsmith, p. 279.
5. PARSSINEN, T. M. (1977) Mesmeric performers, *Victorian Studies,* **21**, 87–104.
6. STORER, H. (1845) Mesmerism in disease, 2nd. ed., London, Bailliere.
7. *Bristol Mercury*, 29 May 1847; 30 December 1848.
8. *Bristol Times*, 2 June 1849.
9. *Bristol Mercury*, 19 July 1851.
10. Last meeting of the Bristol Mesmeric Institute, *Zoist,* 1853, **10**, 158–161 and p. 158 f.n.
11. ESTLIN, J. B. (1843) An address to the members of the medical profession of Bath and Bristol on mesmerism, London, Henry Renshaw.
12. ESTLIN, J. B. (1845) Remarks on mesmerism in 1845, *Provincial Medical & Surgical Journal,* 513–517.
13. *Bristol Mercury*, 3 May 1851.
14. MELLOR, H. E. (1976) Leisure and the changing city, 1870–1914, London, Routledge & Kegan Paul, pp. 42–48.
15. BROWN, P. S. (1982) Herbalists and medical botanists in mid-nineteenth century Britain, with special reference to Bristol, *Medical History,* **26**, 405–420.
16. *Bristol Times and Felix Farley's Bristol Journal,* 5 January 1856.
17. *Bristol Mercury*, 22 May 1847.
18. *ibid.*, 26 June 1847.
19. *ibid.*, 19 August 1848.
20. PARSSINEN, T. M. (1979) Professional deviants and the history of medicine: medical mesmerism in Victorian Britain, in R. Wallis ed. On the margins of science: the social construction of rejected knowledge, *Sociological Review Monograph,* **27**, 103–120.
21. *Times*, 30 November 1841, p. 21.
22. *Medical Galvanism,* vol. 110, n.d., published De l'Huynes, London.
23. *Med.TimesGaz.*, 1855, **11**, 102.
24. *Dr. Skelton's Botanic Record and Family Herbal,* 5 August 1854.
25. TURRELL, W. J. (1921) Three electrotherapists of the eighteenth century: John Wesley, Jean Paul Marat and James Graham, *Ann.med.Hist.,* **3**, 361–367.
26. BROWN, P. S. (1980) The providers of medical treatment in mid-nineteenth century Bristol, *Medical History,* **24**, 297–314.
27. *Clifton Chronicle*, 31 August 1852.
28. Book review, *Lancet,* 1855, **2**, 12.
29. *Lancet,* 1847, **1**, 287.
30. BIRD, G., (1849) Lectures on electricity and galvanism, in their physiological and therapeutical relations, London, Longman, Brown, Green & Longman, pp. 123–124.
31. SYMONDS, J. A., (1868) Therapeutic memoranda, *Brit.med.J.,* **1**, 448–449.
32. SPENDER, J. K., (1982) Thirty years of therapeutic progress, Bath, Curtis.
33. SYMES, J. O. (1930) A short history of the Bristol General Hospital, Bristol, John Wright & Sons, p. 98.
34. PARKER, G. (1904) Notes on some cases from an electrical department, *Bristol Med.-Chir.J.,* **22**, 112–120.
35. *Lancet*, 1842–3, **2**, 622–623.
36. Unqualified electricians, *Lancet,* 1859, **1**, 153 and 205.
37. *Times,* 8 June 1861, p. 11d; II June 1861, p. 11b.
38. *Bristol Mercury*, 4 January 1851.
39. TYLEE, J. P. (1848) Practical observations on galvanism, electricity and electromagnetism, as employed in the cure of disease, Bath, the author, p. 35.

